# Impaired autophagy‐mediated macrophage polarization contributes to age‐related hyposalivation

**DOI:** 10.1111/cpr.13714

**Published:** 2024-07-14

**Authors:** Zhili Xin, Rongyao Xu, Yangjiele Dong, Shenghao Jin, Xiao Ge, Xin Shen, Songsong Guo, Yu Fu, Ping Zhang, Hongbing Jiang

**Affiliations:** ^1^ Department of Oral and Maxillofacial Surgery, Affiliated Hospital of Stomatology Nanjing Medical University Nanjing China; ^2^ State Key Laboratory Cultivation Base of Research, Prevention and Treatment for Oral Diseases Nanjing Medical University Nanjing China; ^3^ Jiangsu Province Engineering Research Center of Stomatological Translational Medicine Nanjing Medical University Nanjing China

## Abstract

Age‐related dysfunction of salivary glands (SGs) leading to xerostomia or dry mouth is typically associated with increased dental caries and difficulties in mastication, deglutition or speech. Inflammaging‐induced hyposalivation plays a significant role in aged SGs; however, the mechanisms by which ageing shapes the inflammatory microenvironment of SGs remain unclear. Here, we show that reduced salivary secretion flow rate in aged human and mice SGs is associated with impaired autophagy and increased M1 polarization of macrophages. Our study reveals the crucial roles of SIRT6 in regulating macrophage autophagy and polarization through the PI3K/AKT/mTOR pathway, as demonstrated by generating two conditional knock out mice. Furthermore, triptolide (TP) effectively rejuvenates macrophage autophagy and polarization via targeting this pathway. We also design a local delivery of TP‐loaded apoptotic extracellular vesicles (ApoEVs) to improve age‐related SGs dysfunction therapeutically. Collectively, our findings uncover a previously unknown link between SIRT6‐regulated autophagy and macrophage polarization in age‐mediated hyposalivation, while our locally therapeutic strategy exhibits potential preventive effects for age‐related hyposalivation.

## Graphical abstract

In this study, we demonstrated impaired autophagy and polarization of macrophage in aged mouse and human salivary gland with age‐related changes. Furthermore, we further validate the in vivo role of SIRT6‐mediated PI3K/AKT/mTOR signal pathway in controlling salivary gland function.

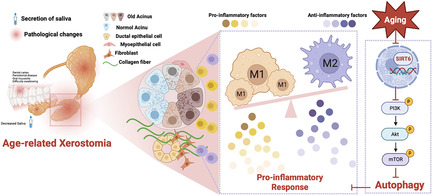

## 1 INTRODUCTION

The primary function of salivary glands (SGs) is the production and secretion of saliva, which plays a crucial role in maintaining oral health. Approximately 95% of saliva originates from three major SGs, namely the submandibular gland, parotid gland and sublingual gland, while the remaining 5% is secreted by minor SGs. Various factors affect saliva secretion, among which ageing is one of the important factors.^1^ The elderly population often presents with a clinical symptom of age‐related hyposalivation, characterized by a significant reduction in both unstimulated and stimulated salivary flow.^2^ When there is a reduction in saliva production, the initial indication is the presence of dry mouth (xerostomia). Approximately 25% of the elderly individuals suffer from xerostomia and associated complaints.^3^ Importantly, xerostomia can also heighten the susceptibility to dental caries, periodontal diseases and oral infections such as candidiasis. Therefore, comprehending the underlying mechanisms of age‐related SGs dysfunction is crucial for strategizing effective therapeutic regimens to prevent xerostomia and simultaneously enhance the quality of life.

With advancing age, SGs undergo increased fibro‐adipose alternation but decreased acinar cells.^4^ In aged SGs, the degeneration of acinar cells associated with ageing is accompanied by ductal dilation, indicating that a prolonged inflammatory microenvironment contributes to SGs dysfunction and hyposalivation.^5^ Macrophages play a crucial role in age‐related inflammation and tissue repair through their involvement in pro‐inflammatory classical M1 activation or anti‐inflammatory alternative M2 activation.^6^ Interestingly, macrophages have been implicated in the paradoxical activation of basal chronic inflammatory states among elderly individuals, suggesting potential variations in macrophage polarization characteristics specific to different organs such as salivary glands.^7^ However, the physiological characteristics and functions of macrophages during SGs ageing remain incompletely elucidated.

Macrophages initiate a range of actions in response to ageing induced by the accumulation of oxidative damage, regulating glycolytic and mitochondrial metabolism, including nicotinamide adenine dinucleotide (NAD^+^).^8^ Our previous studies have demonstrated that SIRT6, an NAD^+^‐dependent histone deacetylase, transcriptionally governs macrophage regulation in glucose metabolism and inflammatory homeostasis.^9^, ^10^ Autophagy is a prevalent cellular mechanism by which cytoplasm and organelles are enclosed within double‐membrane vesicles and subsequently transported to lysosomes for degradation, facilitating the potential recycling of resulting macromolecules.^11^ Among its various regulatory functions such as maintaining cellular homeostasis, promoting anti‐ageing effects and contributing to development processes, autophagy has been acknowledged for its significant role in controlling the homeostasis of SGs.^12^, ^13^ By targeting autophagy‐related proteins like ATG5, ATG7 and BECN1 in response to ageing, autophagy regulates macrophage polarization.^14^, ^15^ Therefore, we propose a hypothesis that age‐induced hyposalivation may be attributed to autophagy‐mediated macrophage polarization leading to structural and functional abnormalities in SGs.

Our study reveals a correlation between age‐related hyposalivation and abnormal macrophage polarization and autophagy in both human and mice SGs. By generating macrophage‐specific SIRT6 and ATG5 knockout mice, we confirm that SIRT6 targets autophagy‐mediated macrophage polarization through the PI3K/AKT/mTOR pathway. Additionally, triptolide (TP), an active component of the Chinese medicinal herb, can regulate macrophage autophagy and polarization. Finally, we demonstrate the efficacy of locally delivered of apoptotic extracellular vesicles (ApoEVs) loaded with TP in preventing age‐related dysfunction of SGs. These findings suggest that targeting macrophages may be a potential preventive and therapeutic strategy for age‐related hyposalivation.

## 2 RESULTS

### 2.1 Hyposalivation in aged SGs accompanied with abnormal autophagy and polarization of macrophages

The secretion function of human SGs was analysed using a weighing method, revealing that the unstimulated whole salivary flow rate (UWS) in the older group was significantly lower compared to the younger group. However, no significant differences were observed in the stimulated whole salivary flow rate (SWS) (Figure S1A). Since UWS primarily reflects the secretion function of submandibular glands (SMG),^16^ we opted to utilize SMG for our subsequent assay. Aged SGs exhibited a reduction in acinar area (Figure S1B,I), along with a significant decrease in the expression of aquaporin 5 (AQP5), an essential water transport ion channel,^17^ (Figure S1C,J). Additionally, an increased number of SA‐β‐gal positive cells and a higher proportion of apoptotic cells were observed in the elderly group (Figure S1D,E,K,L). To assess the impact of ageing on macrophage autophagy and polarization, we conducted immunofluorescent double‐labelled staining and observed a reduced presence of CD68^+^ATG5^+^ cells in aged SGs, indicating diminished autophagy activity in aged macrophages (Figure S1F,M). Furthermore, an increased CD68^+^CD86^+^ M1 macrophages and a decreased number of CD68^+^CD206^+^ M2 macrophages were evident in aged SGs, although no statistical difference was found in the abundance of in M2 macrophages between the two groups (Figure S1G,H,N,O).

**FIGURE 1:**
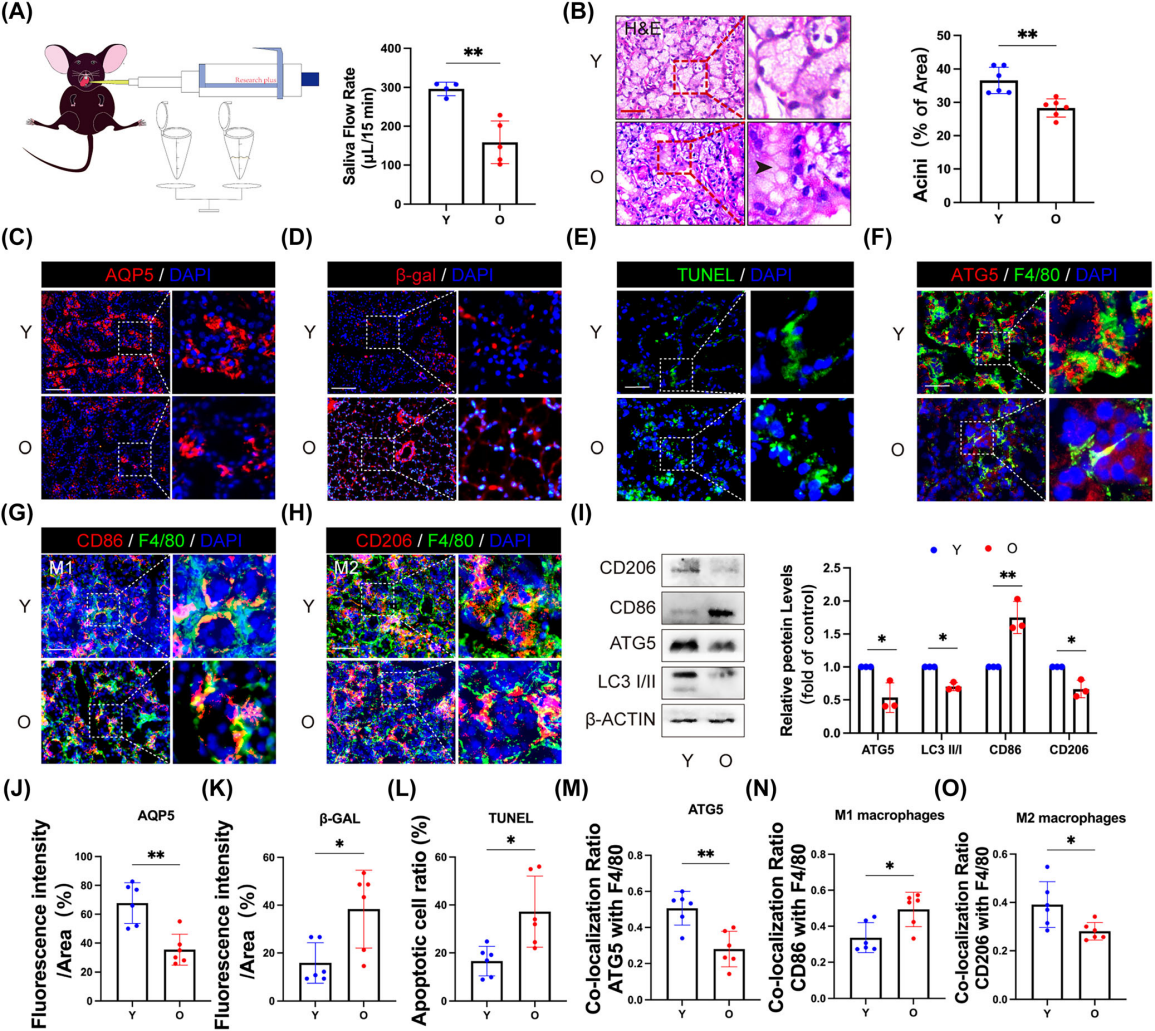
Hyposalivation of the aged SGs accompanied with abnormal autophagy and polarization of macrophages. (A) Changes of SFR (μL/min) in the young (Y; *n* = 4) and the aged (O; *n* = 5) C57BL/6 mice group. (B) The histological section stained with H&E showed the ageing‐related changes in the SG structure of mice. Black arrowhead indicated atrophic acini. *n* = 6. Bar: 200 μm. (C) Immunofluorescent microscopy of SG sections for AQP5 in mice. *n* = 6. Bar: 100 μm. (D) Representative images of β‐GAL (red) staining in cryosections from mice SG sections. *n* = 6. Bar: 100 μm. (E) TUNEL staining in Y and O groups. *n* = 6. Bar: 100 μm. (F and G) The Immunofluorescence staining images of ATG5 (red), F4/80 (green) and DAPI (blue) labelled macrophages in SGs. *n* = 6. Bar: 200 μm. (H) The immunofluorescence staining to identify M1‐like macrophages (F4/80^+^ CD86^+^) and M2‐like macrophages (F4/80^+^ CD206^+^). *n* = 6. Bar: 200 μm. (I) Autophagy‐related protein (LC3 II/I and ATG5) and macrophage type marker (CD86 and CD206) were analysed by Western blot. β‐ACTIN served as a loading control. *n* = 3. (J–O) Results are presented as the mean ± SD by one‐way ANOVA followed with Tukey multiple comparisons tests or unpaired 2‐tailed Student *t*‐tests. *p < 0.05; **p < 0.01.

To further investigate the changes in salivary secretion function in aged mice, we conducted measurements of salivary flow rate (SFR) and observed a significant reduction of SFR in aged mice (Figure 1A). Consistent with the findings in human SGs, there was a notable decrease in both the proportion of acinus area (Figure 1B) and the expression of AQP5 (Figure 1C,J) among aged mice. Additionally, there was a marked increase in the number of SA‐β‐gal‐positive cells with age (Figure 1D,K). We also identified an elevated presence of apoptosis cells within aged SGs (Figure 1E,L). Furthermore, autophagy activity decreased among macrophages within SGs of aged mice (Figure 1F,M), along with a shift towards polarization more inclined to M1 rather than M2 macrophages (Figures 1G,H,N,O and S2A). Macrophages within the tissue can be categorized into two types: tissue‐resident macrophages (rMφs) originating from the yolk sac and the foetal liver; infiltrating macrophages (iMφs) derived from bone marrow.^18^ Consistent with previous findings,^19^, ^20^ our data showed a significantly lower proportion of CD45^+^ CD11C^+^ CD11B^−^ F4/80^+^ labelled rMφs compared to that of CD45^+^ CD11C^−^ CD11B^+^ F4/80^+^ labelled iMφs in SGs (Figure S2B). Consequently, we isolated macrophages from bone marrow for cytological investigations. Notably, aged BMDMs exhibited decreased expression level of ATG5 and LC3 II/I ratio (Figure 1I). Additionally, an up‐regulation of CD86 and down‐regulation of CD206 were observed in aged BMDMs (Figure 1I), further suggesting that aberrant autophagy and polarization contribute partially to age‐related changes in SGs.

### 2.2 Macrophage‐specific SIRT6 knockout leads to age‐related dysfunction of SGs

**FIGURE 2:**
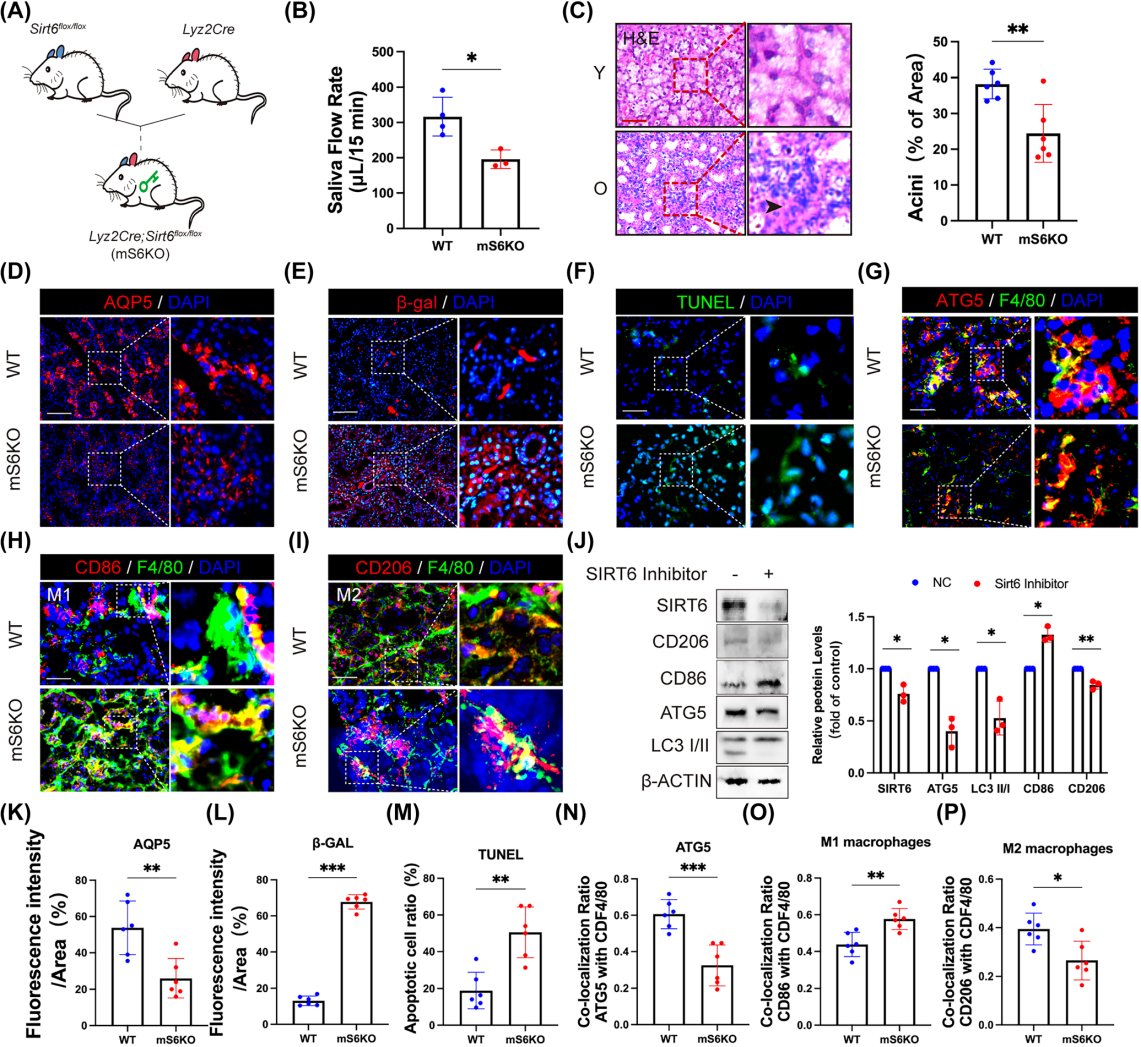
Macrophage‐specific SIRT6 knockout leads to age‐related dysfunction of SGs. (A) Generation of macrophage conditional knockout SIRT6 mice (*Lyz2Cre; SIRT6* ^*f/f*^ mice; mS6KO) using *Lyz2Cre* and *SIRT6* ^*f/f*^ mice. (B) Quantitative analysis of SFR (μL/min) in the wild type (WT; *n* = 4) and mS6KO group (*n* = 3). (C) Histological changes in the SGs of mS6KO mice observed by H&E staining. Black arrowhead indicated atrophic acini. *n* = 6. Bar: 200 μm. (D) The expressions of AQP5 in the WT and mS6KO groups were determined by IHF. *n* = 6. Bar: 100 μm. (E) The Immunofluorescence staining was detected using anti‐β‐gal antibodies. *n* = 6. Bar: 100 μm. (F) The images of TUNEL‐positive cells were captured by a fluorescence microscope. *n* = 6. Bar: 200 μm. (G) Representative images of ATG5 (red), F4/80 (green) and DAPI (blue) labelled macrophages in SG sections. *n* = 6. Bar: 200 μm. (H and I) IHF staining of M1/M2 macrophage in SGs from mice. *n* = 6. Bar: 200 μm. (J) The expression of LC3 II/I, ATG5, CD86 and CD206 in the mice bone marrow‐derived macrophages (BMDMs) after SIRT6 Inhibitor (OSS_128167) treatment analysed by Western blot. β‐ACTIN served as a loading control. *n* = 3. (K–P) Results are presented as the mean ± SD by one‐way ANOVA followed with Tukey multiple comparisons tests or unpaired 2‐tailed Student *t*‐tests. *p < 0.05; **p < 0.01; ***p < 0.001.

To further investigate the impact of ageing on macrophage autophagy and polarization in SGs, we generated mice with a specific knockout of SIRT6 in macrophages (mS6KO) by crossing *Lyz2Cre* mice and *SIRT6* ^*flox/flox*^ mice (Figure 2A). As anticipated, the expression of SIRT6 in SGs macrophages from mS6KO mice was deficient (Figure S3A,B). Analysis of SFR revealed impaired salivary secretion function in mS6KO mice compared to wild‐type (WT) mice (Figure 2B). Additionally, the mS6KO mice exhibited reduced acinus area and dilated ducts (Figure 2C). Using immunofluorescence analysis, we observed a reduction in the expression of AQP5 (Figure 2D,K) and an increase in senescent and apoptotic cells (Figure 2E,F,L,M) within the SGs of mS6KO mice. These findings indicate that SIRT6 is essential for maintaining SGs homeostasis and function in macrophages, and its deficiency contributed to age‐related dysfunction of SGs. Furthermore, mS6KO mice not only exhibited a significant decrease in ATG5 level (Figure 2G,N) but also showed a tendency towards decreased anti‐inflammatory M2 macrophages and increased pro‐inflammatory M1 macrophages within the SGs' macrophage population (Figure 2H,I,O,P). To further investigate the impact of SIRT6 deficiency on autophagy and polarization of macrophages in vitro, we treated bone marrow‐derived macrophages with OSS_128167 (SIRT6 inhibitor). In conjunction with the decline of SIRT6, the levels of autophagy marker proteins, such as ATG5 and LC3 II/I, were also observed to decrease upon exposure to the SIRT6 inhibitor (Figure 2J). Simultaneously, the SIRT6 inhibitor diminished M2 biomarkers while elevating M1 biomarkers (Figure 2J). These findings suggest that deletion of SIRT6 suppresses macrophage autophagy and subsequently disrupts polarization regulation, thereby exacerbating age‐related degeneration of SGs.

### 2.3 SITR6 regulates autophagy and polarization of macrophages via PI3K/AKT/mTOR pathway

**FIGURE 3:**
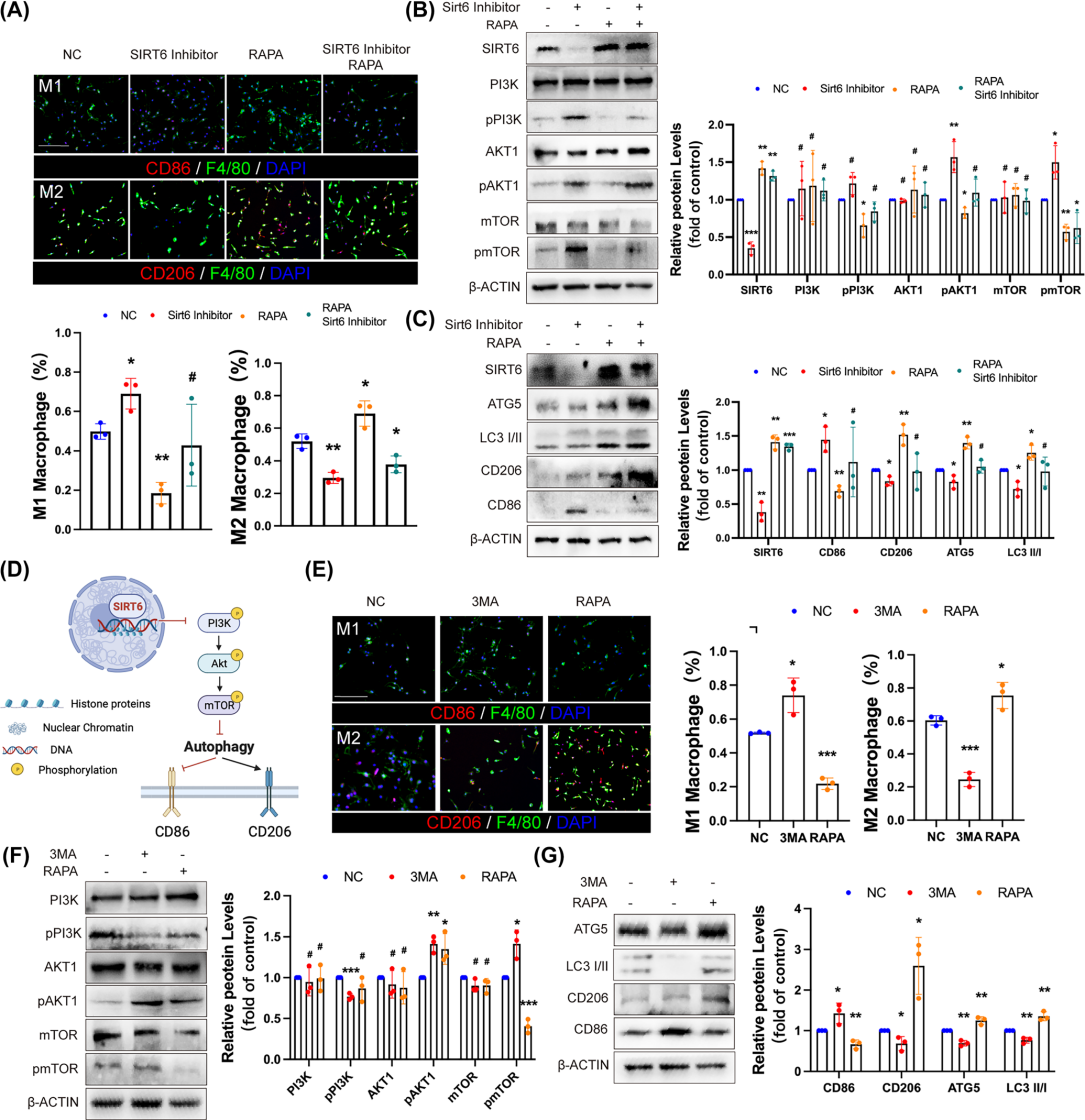
SITR6 regulates autophagy and polarization of macrophages via PI3K/AKT/mTOR pathway. (A and E) Immunocyte fluorescence for M1 (CD86) and M2 (CD206) macrophage markers with SIRT6 Inhibitor, 3‐Methyladenine (3MA) and Rapamycin (RAPA) treatment. *n* = 3. Bar: 200 μm. (B and F) Levels of proteins involved in PI3K/AKT/mTOR pathways concluding PI3K, pPI3K, AKT1, pAKT1, mTOR, pmTOR and β‐ACTIN with SIRT6 Inhibitor, 3MA and RAPA treatment. (C and G) Western blot analysis showing LC3 II/I, ATG5, CD86, CD206 and β‐ACTIN in the BMDMs after SIRT6 Inhibitor, 3MA and RAPA treatments. *n* = 3. (D) Schematic diagram indicated that SITR6 regulates autophagy to affect macrophage polarization via PI3K/AKT/mTOR pathway. Results are presented as the mean ± SD by one‐way ANOVA followed with Tukey multiple comparisons tests or unpaired 2‐tailed Student *t*‐tests. *p < 0.05; **p < 0.01; ***p < 0.001; #*p* > 0.05.

To elucidate the underlying molecular mechanisms by which SIRT6 governs autophagy and polarization of macrophages, we initially showed that BMDMs exhibited a tendency towards M1 polarization in the presence of a SIRT6 inhibitor, while M2 polarization was observed in the Rapamycin (RAPA) group (Figure 3A). Furthermore, co‐treatment with both the SIRT6 inhibitor and RAPA resulted in an increase in M1 macrophages and a decrease in M2 macrophages compared with the RAPA group alone (Figure 3A). SIRT6 has been reported to inhibit the PI3K/AKT/mTOR pathway by inhibiting AKT activation, affecting PI3K activity and regulating glucose metabolism.^21^ Consistently, our findings also demonstrated that the SIRT6 inhibitor activated the PI3K/AKT/mTOR pathway, whereas RAPA not only inhibited this pathway but also reversed the effect induced by the SIRT6 inhibitor (Figure 3B). Western blot analysis further revealed that treatment with the SIRT6 inhibitor down‐regulated expression levels of autophagy‐related factors LC3 II and ATG5 while concurrently up‐regulating expression levels of CD86 as an M1 biomarker. The results in the RAPA group, however, demonstrated contrasting findings (Figure 3C). Collectively, SIRT6 potentially regulates autophagy and macrophage polarization through the PI3K/AKT/mTOR pathway (Figure 3D). To further validate this pathway, we employed the PI3K inhibitor 3‐methyladenine (3MA), or mTOR inhibitor RAPA and observed that 3MA promoted M1 macrophage polarization while inhibiting M2 macrophage polarization; conversely, opposite outcomes were observed in the RAPA group (Figure 3E). Additionally, macrophages treated with 3MA exhibited activation of the PI3K/AKT/mTOR pathway, whereas contrasting results were showed in the RAPA group (Figure 3F). Autophagy‐related proteins ATG5 and LC3 II/I decreased in the presence of 3MA along with an increased M1 polarization; however, contrary results were observed in both the RAPA and Baf A1 groups (Figures 3G and S5A,B). These results suggest that SITR6 partially regulates autophagy and macrophage polarization via the PI3K/AKT/mTOR pathway.

### 2.4 Autophagy‐related gene knockout mice show age‐related characterization of SGs

**FIGURE 4:**
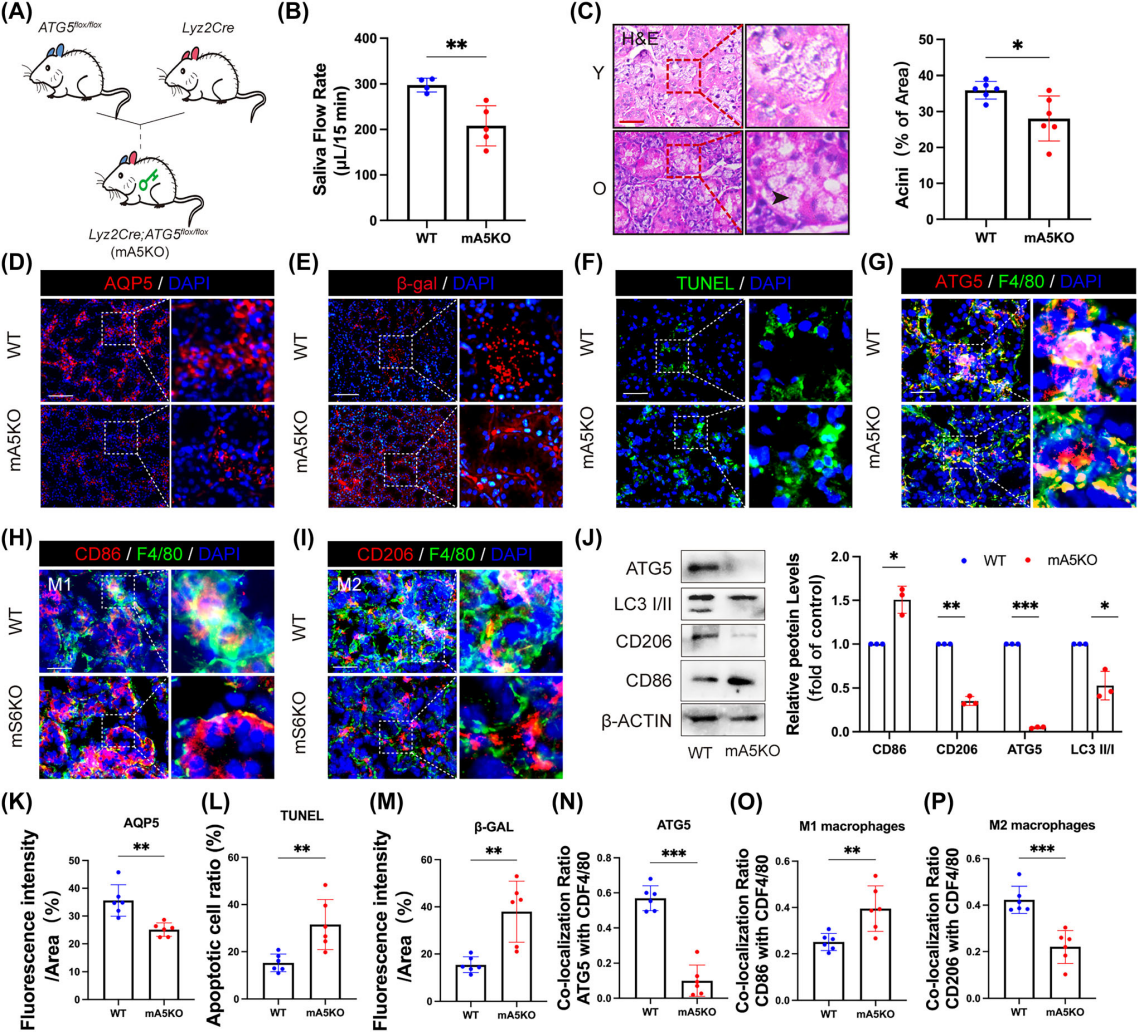
Autophagy‐related gene knockout mice show age‐related characterization of SGs. (A) Generation of macrophage conditional knockout ATG mice (*Lyz2Cre;Atg5* ^*f/f*^ mice; mA5KO) using *Lyz2Cre* and *ATG5* ^*f/f*^ mice. (B) The SFR (μL/min) of mA5KO (*n* = 5) mice was reduced compared with WT (*n* = 4). (C) Representative image of H&E stained showed the SG of mA5KO mice. Black arrowhead indicated atrophic acini. *n* = 6. Bar: 200 μm. (D) IHF showed levels of AQP5 protein in the SG tissue from WT and mA5KO mice. *n* = 6. Bar: 100 μm. (E) IHF using a monoclonal antibody to β‐Gal shown are photomicrographs of the resulting cells expressing β‐Gal (red). *n* = 6. Bar: 100 μm. (F) TUNEL assay of SG tissues. *n* = 6. Bar: 200 μm. (G) Expression of ATG5 (red) in F4/80 (green) positive cells represented macrophages undergoing autophagy in the SG tissue. *n* = 6. Bar: 200 μm. (H and I) IHF analysis of polarized macrophages such as CD86 (M1) and CD206 (M2). *n* = 6. Bar: 200 μm. (J) Western blot analysis of LC3 II/I, ATG5, CD86 and CD206 protein in the BMDMs from mA5KO and WT mice. β‐ACTIN served as a loading control. *n* = 3. (K–P) Results are presented as the mean ± SD by one‐way ANOVA followed with Tukey multiple comparisons tests or unpaired 2‐tailed Student *t*‐tests. *p < 0.05; **p < 0.01; ***p < 0.001.

To further explore the role of macrophage autophagy in SGs, we generated *Lyz2Cre;ATG5* ^*f/f*^ mice (mA5KO) to examine its impact (Figure 4A). We observed a lower SFR was lower in mA5KO mice compared to WT mice, indicating impaired function of SGs (Figure 4B). Histological staining showed a significant decline in acinar area in mA5KO mice, suggesting structural changes within the SGs (Figure 4C). Furthermore, mA5KO mice exhibited noticeably decreased expression of AQP5 (Figure 4D,K). Additionally, there was an increased presence of SA‐β‐gal positive cells indicative of cellular senescence and apoptotic cells within the SGs of mA5KO mice compared to WT controls (Figure 4E,L and F,M). The results suggest that the clearance of senescent and apoptotic cells is impaired due to the deletion of ATG5. Consistent with this, macrophages in SGs from mA5KO mice exhibited deficient expression of ATG5 (Figure 4G,N). Furthermore, ATG5 deficiency led to an increased in F4/80^+^ CD86^+^ M1 macrophages (Figure 4H,O), while there was a decrease in F4/80^+^ CD206^+^ M2 macrophages in SGs (Figure 4I,P). To determine the impact of macrophage‐specific ATG5 deletion on macrophages polarization, we isolated BMDMs from mA5KO mice and identified low expression of the LC3 II and CD206, along with high expression level of CD86 (Figure 4J). Together, these findings demonstrate that conditional knockout of ATG5 in macrophages leads to hyposalivation and promotes pro‐inflammatory M1 macrophage polarization in SGs.

### 2.5 TP mediates autophagy‐mediated macrophage polarization via PI3K/AKT/mTOR

**FIGURE 5:**
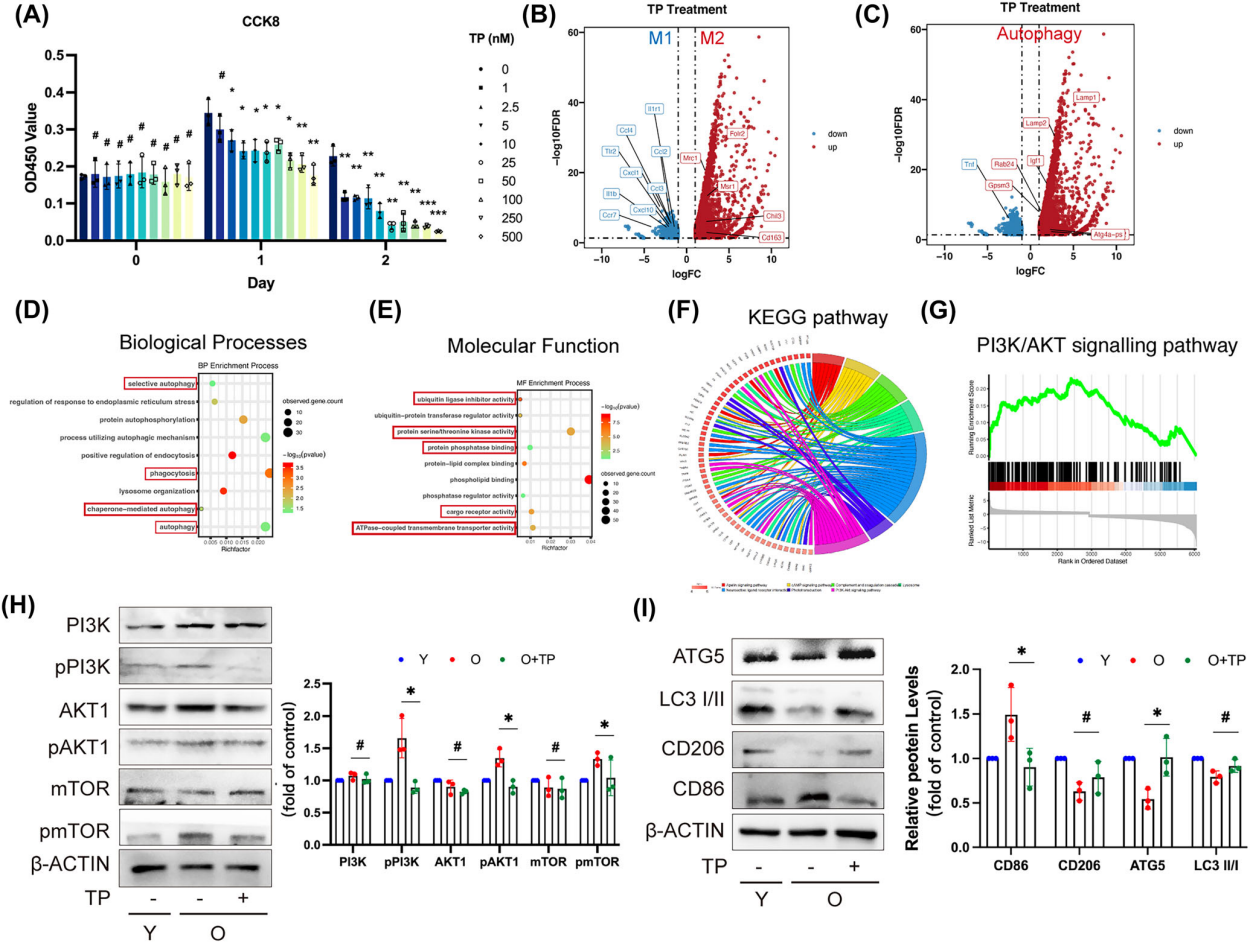
TP mediates autophagy‐mediated macrophage polarization via PI3K/AKT/mTOR. (A) Cell counting kit‐8 (CCK‐8) assay showing cell viability (OD450 value) with various concentrations of free TP treatment for 1 or 2 days. *n* = 3. (B) Volcano plot showing the distribution of up‐regulated genes (red; M2 macrophages‐related gene; dots in the right box) or down‐regulated genes (blue; M1 macrophages‐related gene; dots in the left box) in BMDMs with TP treatment group (Treatment group) compared that without TP treatment (Control group). (C) Volcano plot showing the autophagy‐related distribution of up‐regulated genes (red; dots in the right box) or down‐regulated genes (blue; dots in the left box) compared Control with the Treatment group. (D and E) GO enrichment results of differentially expressed genes between Control and Treatment groups were displayed in the bubble plots and included biological process (BP) and molecular function (MF). The bubble colour represents the *p*‐value, and the bubble size represents the number of genes in the relevant pathway (see the legend on the right‐hand side). (F) KEGG pathway cluster analyses. (G) GSEA plots of DEGs enriched in the Treatment group. The data analysed based on the GO of the biological process database was mainly enriched in the PI3K/Akt signalling pathway by GSEA analysis. (H) Western blot analysis of PI3K/AKT/mTOR signalling pathway‐related proteins concluding PI3K, pPI3K, AKT1, pAKT1, mTOR, pmTOR and β‐ACTIN in each group (young BMDMs [Y]; old BMDMs [O]; old BMDMs with TP treatment [O + TP]). *n* = 3. (I) Western blot detected the protein levels of ATG5, LC3 II/I, CD86 and CD206 in each group. *n* = 3. Results are presented as the mean ± SD by one‐way ANOVA followed with Tukey multiple comparisons tests or unpaired 2‐tailed Student *t*‐tests. *p < 0.05; **p < 0.01; ***p < 0.001; #*p* > 0.05.

Autophagy‐mediated anti‐inflammatory effects of TP^22^, ^23^ have prompted us to investigate whether TP prevents age‐related dysfunction of SGs by mediating autophagy and macrophages polarization. Firstly, a safe concentration of 2.5 ng/mL TP was determined through the CCK8 assay (Figure 5A). Secondly, we performed RNA sequencing (RNA‐seq) and analysed the differentially expressed genes (DEGs) regulated by M1/M2 macrophages based on the RNA‐seq data analysis. The expression levels of M1 macrophage‐related marker genes including CCL2, IL‐1b, CCR7, CCL4, CD64, CXCL10, IL‐15R, IL‐1R‐1, TLR2, Ccl3, Cxcl1, Cxcl10 and FcRI were down‐regulated while those of M2 macrophage‐associated marker genes such as CD163, CD206, Chil3, FOLR2, Mrc1, MSR1, Tie‐2, CD36 and CD32 were up‐regulated (Figure 5B). Meanwhile, we analysed the data of the autophagy‐regulated DEGs involved in autophagy elongation including ATG9, GABARAP, GABRRAPL1(ATG8l), GABARAPL2, ATG4 and p62 which were found to be up‐regulated (Figure 5C). Interestingly, the expression levels of LAMP1, LAMP2 and Rab24 involved in the fusion of autophagosome and lysosome were also up‐regulated (Figure 5C). In addition, autophagy transport‐related genes involved GPSM3, Hsp90aa1, PIK3C3 and Rab24 as well as autophagy regulating‐related gene App were up‐regulated (Figure 5C). The enrichment analysis of up‐regulated DEGs for biological processes (BP) ontology terms revealed their association with various aspects of autophagy including selective autophagy, phagocytosis and chaperone‐mediated autophagy (Figure 5D). The molecular function (MF) of Gene Ontology (GO) analysis showed that the up‐regulation of DEGs was mainly related to the process of autophagy including ubiquitin binding, protein kinase activity, protein phosphatase activity and ATPase activity (Figure 5E). The PI3K/AKT signalling pathway was identified through analysis using the KEGG pathway database (Figure 5F). Subsequently, a GSEA analysis was conducted to examine the down‐regulated genes in the PI3K/AKT/mTOR pathway along with the up‐regulated genes (Figure 5G). These findings suggest that the PI3K/AKT/mTOR signalling pathway plays a crucial role in regulating macrophage polarization during TP treatment. Furthermore, we validated this by observing an inclination towards M2 macrophage polarization accompanied by enhanced autophagy in BMDMs from aged mice treated with TP (Figure 5I). Notably, phosphorylation levels of PI3K, AKT1 and mTOR decreased in aged BMDMs following TP treatment (Figure 5H). Taken together, these results suggest that TP promotes the autophagy and M2 polarization of aged macrophages by inhibiting the PI3K/AKT/mTOR signal pathway.

### 2.6 TP‐loaded ApoEVs prevent age‐related SGs dysfunction

**FIGURE 6:**
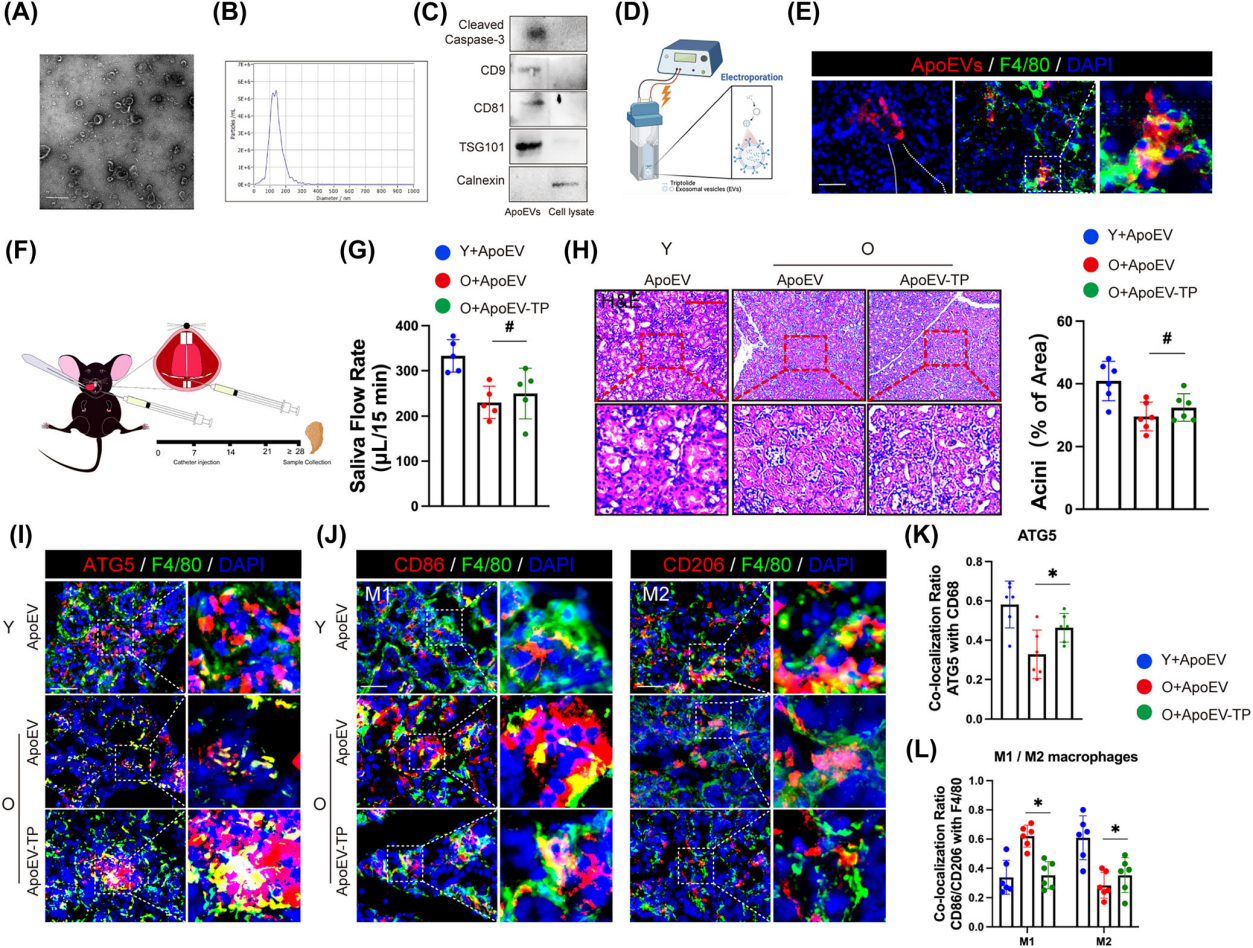
TP‐loaded ApoEVs prevent age‐related SGs dysfunction. (A) Morphology of exosomes was observed using transmission electron microscopy (TEM). Bar: 500 nm. (B) Nanoparticle tracking analysis (NTA) of exosomes isolated from MC3T3‐e1 cell. The concentration is displayed as particles/mL. (C) Expression levels of CD9, TSG101, cleaved caspase‐3 and CD81 the apoptotic extracellular vesicles (ApoEVs) positive markers, and Calnexin the exosomes‐negative markers were observed by Western blot. The cell lysate was used as a control. (D) Loading TP into ApoEVs by electroporation. (E) Confocal images showed that the macrophages uptake ApoEVs in submandibular glands. ApoEVs were labelled with Dil (red) and macrophages were labelled with F4/80 (green). The white dashed line represents the submandibular gland duct. Bar: 100 μm. (F) Schematic diagram of the animal experiment. Construction of old mice with ApoEVs injection through submandibular gland duct or not once per week for 4 weeks. (G) The SFR (μL/min) of old mice with TP‐loaded ApoEVs treatment (O + ApoEV‐TP) was modestly increased compared to that with ApoEVs treatment (O + ApoEV). *n* = 5. (H) Analysed the percentage of acinar area in each group (young mice with ApoEVs treatment [Y + ApoEV]; O + ApoEV; O + ApoEV‐TP). *n* = 6. Bar: 200 μm. (I) Expression of autophagy in macrophage was confirmed by F4/80 (green), ATG5 (red) and DAPI (blue) immunostaining. (J) IHF analysis of polarized macrophage population using phenotypic surface markers such as CD86 (M1) and CD206 (M2). *n* = 6. Bar: 200 μm. (K and L) Results are presented as the mean ± SD by one‐way ANOVA followed with Tukey multiple comparisons tests or unpaired 2‐tailed Student *t*‐tests. *p < 0.05; #*p* > 0.05.

As a drug‐delivery system, ApoEVs demonstrate potential therapeutic applications.^24^ To determine whether TP‐loaded ApoEVs could prevent age‐related SGs dysfunction, we initially fabricated ApoEVs (Figure 6A). The size distribution of the ApoEVs was analysed using nanoparticle tracking analysis (Figure 6B). Cleaved caspase‐3 (apoptosis markers), CD9, CD63 and TSG101 (exosomal markers) exhibited high expression levels within the ApoEVs, while calnexin (a cytosolic marker) was minimally detectable (Figure 6C). We loaded TP into exosomes via electroporation (Figure 6D), and delivered TP‐loaded ApoEVs into the SMG of 18‐month‐old mice through ducts once a week for 1 month (Figure 6F). Considering the dominant phagocytes of macrophages in the salivary gland (Figure S4A), we confirmed the feasibility of this therapeutic approach shown as a FITC‐labelled F4/80 macrophages co‐localized with Dil‐labelled ApoEVs after delivery (Figure 6E). We observed an increased SFR in aged mice after TP‐loaded ApoEVs treatment despite no statistical difference (Figure 6G). Histological staining revealed a slight increase in acinus area and no obvious organ toxicity in the treatment group compared to the control group (Figure 6H). Importantly, TP‐loaded ApoEVs exhibited potential inhibitory effects on the PI3K/AKT/mTOR signal pathway (Figure S6A–D) while promoting autophagy in macrophage (Figure 6I,K). Meanwhile, the proportion of M1 polarization decreased while there was an increase in the proportion of M2 polarization (Figure 6J,L). Collectively, these findings provide preliminary evidence for the potential utility of TP‐loaded ApoEVs in addressing age‐related Xerostomia.

## 3 DISCUSSION

Xerostomia or the perception of a dry mouth is a potentially debilitating condition that can affect 20% of dental patients, with an increased prevalence in the elderly.^25^ Age‐related structural and functional changes in the SGs may increase the risk of xerostomia. These changes are characterized by a decreased number of acinar cells, increased fibrosis and fatty infiltration.^26^, ^27^ In this study, we observed decreased salivary secretion and age‐related degeneration in both humans and mice. By constructing two genetic mice models, we further confirmed that impaired autophagy‐mediated polarization of macrophages contributed to the dysfunction of aged SGs. Mechanistically, we demonstrated the regulatory mechanism of senescent macrophages through the SIRT6‐targeted the PI3K/AKT/mTOR signalling pathway. Additionally, based on the underlying mechanism, we locally administered TP‐loaded ApoEVs through the salivary duct, thereby revealing their potential in preventing age‐related Xerostomia. Our findings unravel a previously undiscovered connection between autophagy‐mediated macrophage polarization and age‐related SGs dysfunction.

The microenvironment of aged SGs exhibits a chronic inflammatory state characterized by oxidative stress, infiltration of immune cells, proliferation of fibroblasts and increased apoptosis of cells.^5^ Macrophages are crucial component of innate immunity and play a central role in inflammation and host defence, serving as a bridge between innate and adaptive immunity.^28^ In the ageing microenvironment, macrophages display an elevated frequency accompanied by a switch in polarization phenotype leading to excessive cytokine production.^29^, ^30^ Ageing may influence M1/M2 activation and subsequently contribute to changes in macrophage polarization phenotypes.^31^ In our study, we observed an increased presence of CD86‐positive M1 macrophages in aged SGs, which were characterized by high expression levels of pro‐inflammatory cytokines. Conversely, a decreased number of CD206‐positive M2 macrophages were found to be involved in tissue remodelling promotion. M1 macrophages exhibit the ability to release inflammatory cytokines such as TNF‐α and IL‐1β^32^ and generate substantial amounts of oxygen free radicals that can cause damage to cellular structures.^33^ Consequently, the inflammatory and oxidative stress microenvironment in aged SGs thus leads to the apoptosis or death of acinar cells. On the other hand, M2 macrophages typically release anti‐inflammatory^34^ and growth factors, which can potentially attenuate the inflammatory response and facilitate cell proliferation and repair processes.^35^ These findings suggested that compromised macrophage polarization in older individuals may disrupt tissue homeostasis by inducing inflammation within the microenvironment.

SIRT6, a histone deacetylase, has been implicated as a regulator of autophagy^36^ and played a crucial role in driving macrophage polarized towards the M2 phenotype.^37^ Therefore, we focused on identifying SIRT6 due to its obvious down‐regulation in aged BMDMs. Our study revealed that SGs from mS6KO mice exhibited age‐related changes such as acinar atrophy, loss of podocyte structure and fibrosis accompanied by decreased SFR. Notably, there was a significant increase in CD86‐expressing macrophages in mS6KO mice favouring the pro‐inflammatory M1 status consistent with ageing. Apart from the alterations in macrophage polarization, we also observed a decline in macrophage autophagy in the SGs of both aged and mS6KO mice. Furthermore, through the utilization of inhibitors (3MA) and activators (RAPA) targeting autophagy, we found that impaired macrophage autophagy promotes M1 macrophage polarization, while moderate activation of autophagy may facilitate the shift towards an M2 phenotype and alleviate inflammatory responses. Hence, it can be inferred that autophagy plays a crucial role in regulating macrophage polarization in age‐related diseases.

Many studies have consistently demonstrated a strong association between ageing and autophagy in macrophages.^38^, ^39^, ^40^ The up‐regulation of SIRT6 expression inhibits autophagy levels by suppressing the IGF/AKT/mTOR signalling pathway.^41^ By generating a myeloid cell‐specific SIRT6 deficiency mice, we have showed that SIRT6 regulates macrophage autophagy and polarization through the PI3K/AKT/mTOR pathway. Importantly, increased M1 polarization in SGs may contribute to sustained inflammation and age‐related hyposalivation. These findings highlight the crucial role of autophagy in maintaining of SG function. The impaired autophagy in ageing macrophages may partially explain why Xerostomia is more prevalent among older individuals.

Currently, there is no effective treatment for xerostomia. TP, an active component of the Chinese medicinal herb, exerts notable anti‐tumour, immunosuppression and anti‐inflammatory effects.^22^, ^23^ Recent research has also highlighted the potential of triptolide in regulating autophagy^42^, ^43^ and modulating macrophage polarization to exert anti‐inflammatory effects.^44^, ^45^ Considering the targeted delivery and biocompatibility of free TP, we have designed a drug encapsulation carrier by utilizing extracellular vesicles released from apoptotic cells.^46^, ^47^, ^48^ In this study, we utilized UV irradiation of MC3T3e‐1 cells to induce apoptosis as the source of ApoEVs production and administered TP‐loaded ApoEVs into the duct of SGs in aged mice. The infusion of ApoEVs into the SGs could be engulfed by macrophages, thereby restoring impaired autophagy and polarization of macrophages. Simultaneously, ApoEV itself does not interfere with the therapeutic effect of ApoEV‐TP (Figure S7). With a marginal recovery of salivary flow rate, it offers a potential approach to enhance macrophage autophagy in the aged SGs tissue, thus promoting recovery from age‐related SGs hypofunction.

This study unveiled a potential correlation between diminished autophagy and altered macrophage polarization, as well as age‐related changes in SGs. However, the impact of autophagy and macrophage polarization on salivary gland epithelium function was not investigated due to challenges associated with culturing such epithelium. Moreover, there exists a reciprocal regulatory relationship between autophagy and polarization in macrophages; however, this study solely focused on exploring the influence of autophagy on polarization. Furthermore, apart from brain microglia, skin Langerhans cells, pulmonary alveolar macrophages, liver Kupfer cells and heart macrophages,^49^ most macrophages in other tissues are iMφs, while only a minority exhibit rMφ characteristics.^50^ Therefore, we opted to isolate BMDMs instead of employing flow cytometry for the collection of macrophages from salivary glands. These limitations can provide valuable insights for future research in this field.

In conclusion, our study revealed that salivary gland function undergoes age‐related changes in both humans and mice, which are accompanied by impaired macrophage polarization and reduced autophagy. The utilization of two conditional gene knockout animal models confirmed the regulatory role of SIRT6 in macrophage autophagy and its impact on polarization phenotype through the PI3K/AKT/mTOR signalling pathway within the context of ageing. From a therapeutic perspective, considering that TP can effectively restore macrophage autophagy and polarization by targeting this specific pathway, we have developed an approach for modulating macrophage autophagy and polarization via localized delivery of TP‐loaded ApoEVs. This innovative strategy holds potential for managing Xerostomia and other age‐related diseases.

## 4 MATERIALS AND METHODS

### 4.1 Human SMG specimen collection

The human subject protocol was approved by the Ethical Committee Department at the Affiliated Hospital of Stomatology of Nanjing Medical University (Approval No. PJ2021‐154‐001). The study included a total of 12 human SMG subjects, which were divided into two groups (young [25–35 years] and old groups [65‐86 years], with 6 in each group) according to age. The SMG samples were collected from neck dissection surgeries for maxillofacial tumours and the absence of infection or tumour metastasis was confirmed through histological examination. The study participants received a verbal explanation of the nature of the study, and written consent was obtained.

### 4.2 Measurement of salivary flow rate in human

According to the inclusion and exclusion criteria (Table S1), we recruited 13 participants (the young group with 5 and the old group with 8) which were divided based on age. The study participants received a verbal explanation of the nature of the study, and written consent was obtained. Unstimulated whole saliva was collected through the Navazesh method.^51^ Stimulated whole saliva was collected in a modified version of the Shimazaki method.^52^ Saliva was recorded and the salivary flow rate (SFR) was measured in mL/min (Supplementary materials and methods). The results were statistically analysed by asymmetric *t*‐test.

### 4.3 Experimental animals

All animal experimental protocols were approved by the Laboratory Animal Care and Use Committee at Nanjing Medical University (Approval No. IACUC‐2310099). Mice were raised on a 14/10 h light/dark cycle in the Animal Research Center of Nanjing Medical University. *Lyz2Cre* (N000056) and wild‐type C57BL/6 mice were obtained from the Model Animal Research Center of Nanjing University. *SIRT6* ^*flox/flox*^ (017334) and *ATG5* ^*flox/flox*^ female mice (RBRC 02975) were from Jackson Laboratory. To knockout SIRT6 specifically in macrophage cells, *Lyz2Cre* mice were mated with *SIRT6* ^*flox/flox*^ mice to generate *Lyz2Cre;SIRT6* ^*flox/+*^ heterozygous mice. Next, by crossing *Lyz2Cre;SIRT6* ^*flox/+*^ and *SIRT6* ^*flox/flox*^ mice, we obtained *Lyz2Cre;SIRT6* ^*flox/flox*^ mice (mS6KO). *Lyz2cre;ATG5* ^*flox/flox*^ mice (mA5KO) were obtained in the same process. 6–8 weeks mS6KO or mA5KO males were used to analyse the role of SIRT6‐mediated autophagy of macrophage in hyposalivation. To analyse the characteristics of salivary glands during natural ageing, the wild‐type C57BL/6 mice were divided into the young group (3 months old) and the old group (18 months old).

### 4.4 Measurement of salivary flow rate in mice

The resting saliva flow in mice is difficult to measure, so we only measured stimulated salivary flow rate according to the method of Lin's report.^53^ After the animals were anaesthetised, saliva secretion was induced by intraperitoneal injection of pilocarpine (Cat# P6503, Sigma‐Aldrich) at a dose of 5 mg/kg body weight.^53^ Stimulated whole saliva was gravimetrically collected using a 20 μL‐sized pipet tip from the oral cavity, over a 15‐min at room temperature. It should be noted that the head of the mice is turned to one side during the saliva collection process to prevent saliva from blocking the airway and causing suffocation.

### 4.5 Haematoxylin and eosin (H&E) staining

H&E staining was conducted on 4 μm thick tissue sections prepared from formalin‐fixed, paraffin‐embedded (FFPE) tissues. FFPE tissue sections underwent deparaffinized in xylene and then hydrated through graded ethanol and distilled water. Subsequently, the slides were stained with haematoxylin and eosin. After a water rinse, each slide was soaked in Gradient alcohol. The final steps involved in xylene I and xylene II to clear the samples. Photomicrographs were obtained using an ortho‐microscope.

### 4.6 Immunofluorescence (IF) assay

For immunofluorescence analysis, mice SMG tissue was fixed with 4% paraformaldehyde for 48 h and subsequently immersed in a 30% sucrose solution for 24 h. Then, the fixed SMG tissues were embedded in OCT for 4‐μm‐thick slices. These slices were permeabilized with 0.1% Triton X‐100 for 5 min, followed by incubation with goat serum to block nonspecific staining. Primary antibodies, as listed in Table S2, were incubated with SMG tissue overnight at 4°C. After the primary antibodies incubation, the slices were rinsed with PBS and incubated with FITC or Cy3‐labelled secondary IgG at 37°C for 1 h. Finally, sections were counterstained with DAPI and images were captured under a fluorescence microscope (Leica Microsystems, Mannheim, Germany). For immunostaining of paraffin‐embedded human SMG tissues, 4‐μm paraffin sections were deparaffinized, and subjected to antigen retrieval in citrate buffer (pH 7.0). The slides were then blocked in 10% goat serum for 1 h and incubated with primary and secondary antibodies as described above. For immunostaining of BMDMs, cells were fixed in 4% PFA for 15 min, permeabilized with 0.1% Triton X‐100 in PBS for 5 min and stained with the appropriate antibodies as described above.

### 4.7 TUNEL assay

Apoptotic cells in SMG tissue were determined by a commercial kit (Cat# A112, Vazyme). Apoptotic cells were visualized and captured using a fluorescence microscope, where green fluorescence signified positive cells and blue fluorescence indicated the nucleus. TUNEL‐positive cells and total cells were measured with Image J Software.

### 4.8 Bone marrow‐derived macrophage isolation, culture and treatment

Bone marrow flushed from mice femurs and tibias was plated in Dulbecco's modified Eagle medium (DMEM), which was supplemented with 10% heat‐inactivated foetal bovine serum (FBS) and 1% antibiotic‐antimycotic solution. To induce the differentiation of macrophages, we added 25 ng/mL of macrophage colony‐stimulating factor (M‐CSF, Cat# 416‐ML, R&D Systems) every 2 days over 6‐day period. Bone marrow‐derived macrophages (BMDMs) were subsequently stimulated with the reagents listed in Table S3.

### 4.9 Western blotting

The whole‐cell lysate was lysed on ice for 30 min using RIPA buffer (Cat# P0013B, Beyotime) containing 10 mM protease inhibitor. Next, the protein concentrations in the samples were quantified using a Bradford protein assay kit (Cat# P0006, Beyotime). Equal protein concentrations were loaded onto 10–12% sodium dodecyl sulphate‐polyacrylamide gel electrophoresis (SDS‐PAGE) and then transferred to Immobilon‐polyvinylidene difluoride (PVDF) membranes. These membranes were blocked using 5% BSA at 37°C for 2 h and subsequently incubated with primary antibodies at 4°C overnight. Detailed information on the primary antibodies is listed in Table S2. After washing with TBST, the membranes were incubated with secondary peroxidase‐conjugated antibodies for 1 h. Finally, the protein bands were visualized using an ECL detection kit (Cat# P10300, NCM) through a gel imaging system.

### 4.10 Cell counting kit‐8 (CCK8) assay

BMDMs were seeded onto 96‐well plates at a density of 1 × 10^4^ cells per well. Then, various concentrations of TP were introduced, and they were incubated for either 24 or 48 h. Following the incubation period, the media containing TP were carefully removed, and the cells were further incubated at 37°C for 2 h with a CCK8 solution at a concentration of 0.5 mg/mL. The number of viable cells was determined by measuring the optical density at 450 nm (OD450) of the formazan product.

### 4.11 RNA‐seq analysis

Total RNA was isolated using Trizol (Life Technologies) following the manufacturer's instructions. Subsequently, small RNA sequencing libraries were prepared and subjected to high‐throughput sequencing using the MiSeq Illumina platform. The quality assessment reports, both before and after data processing, were generated using FastQC. Following this, the cleaned reads were aligned to a reference genome or transcriptome using alignment algorithms such as Bowtie, STAR or HISAT. After read alignment, transcript assembly and quantification were performed, allowing for the comparison of transcript abundance between different experimental conditions or sample groups. DESeq2 were employed to identify genes exhibiting significant differences. To further elucidate the biological implications, differentially expressed genes were subjected to gene ontology (GO) enrichment analysis, KEGG pathway analysis or other functional annotation tools. The results were then presented through graphical representations, such as volcano plots, heatmaps and gene expression profiles, utilizing R packages such as ‘heatmap’ and ‘ggplot2’.

### 4.12 Isolation of ApoEVs

For the preparation of ApoEVs, the MC3T3‐e1 Cell was subjected to 1 h of UV irradiation in serum‐free αMEM. Then, the medium was replaced with a fresh medium, and the cell culture supernatant was collected 24 and 48 h later to isolate EVs. Briefly, the supernatant underwent a series of centrifugation steps, including 300*g* for 10 min, 2000*g* for 15 min, and 10,000*g* for 30 min, to eliminate cells and debris. Then, ApoEVs were isolated from the supernatant using by ultracentrifugation at 100,000*g* for 70 min at 4°C. Finally, ApoEVs pellets were resuspended with 100 μL PBS and stored at 80°C.

### 4.13 ApoEVs feature analysis

ApoEVs was fixed with an Electron microscope fixative (Cat# G1102, Servicebio) for 30 min. Next, 200‐mesh formvar copper grids were subjected to the Glow Discharge technique for 1 min. 5 μL ApoEVs suspension solution was added to the grids and incubated at 4°C for 1 min. Subsequently, the grids were negatively stained with 1% uranyl acetate for 2 min washed with distilled water, and dried out in the darkness for 10 min under room temperature. Finally, the samples were analysed by TEM at 80 Kv. Nanoparticle‐tracking analysis (NTA) was performed to analyse the size distribution of ApoEVs according to the literature.^54^ In brief, the samples were diluted in a 1:10 ratio with PBS. Then 30‐second videos were recorded, and the particle size was analysed by NTA software (version 3.2NanoSight). We assessed the presence of tetraspanins on ApoEVs surface as well as the expression of apoptosis‐related markers through Western blotting.

### 4.14 Macrophages uptaking ApoEVs in vivo

To detect the uptake of ApoEVs by macrophages in SMGs, we labelled MC3T3‐e1 cells with Dil cell‐labelling solution (Cat# 40718ES50, Yeasen) according to the manufacturer's protocol and collected ApoEVs as described above. After mice anaesthesia, the tongue of mice was lifted to find the opening of the SMG ducts, and Dil‐labelled ApoEVs were administered via an insulin injection needle. Then, the SMG tissues were harvested 24 h post‐injection, embedded in OCT and sectioned into 4 μm thickness. FITC‐labelled F4/80 antibodies were incubated with the SMG tissue at 37°C for 2 h. Subsequently, the slices were rinsed with PBS and stained with DAPI. Finally, fluorescence microscope was employed to capture images immediately.

### 4.15 TP‐loaded ApoEVs treatment

ApoEVs were diluted in PBS at 4°C. The TP at a final concentration of 2.5 ng/mL was added into 100 μL ApoEVs sample and electroporated at 0.150 kV/100 μF under BTX Gemini X2 system. TP‐loaded ApoEVs were injected into mice once per week via ducts of SMGs. After 1 month, SMG samples were harvested for further analysis (*n* = 6 per group).

### 4.16 Data analysis and statistics

We utilized Fiji software to quantify the acini area and positive staining by calculating ‘mean intensity’ of Two‐colour immunofluorescence staining and Pearson's coefficient of Three‐colour immunofluorescence staining. Data are presented as the mean ± SD for all the experiments. Tests for the significance of differences between the groups were performed by the *t*‐test (two groups) or one‐way analysis of variance (one‐way ANOVA) in GraphPad Prism 70 (GraphPad Software, USA). Data with *p* < 0.05 were considered significantly different.

## AUTHOR CONTRIBUTIONS

Z.X. performed the experiments, analysed data and wrote the manuscript. R.X. and S.J. assisted Z.X. with animal studies and the collection of human clinical samples. Y.F. and X.G. collected and analysed the data of RNA‐seq. S.G., P.Z., R.X. and X.S. helped design experiments. Y.D. helped revise manuscripts and perform supplementary experiments after the article was reviewed. H.J. supervised the work and revised the manuscript. All authors read and approved the final manuscript.

## FUNDING INFORMATION

This work was supported by the National Natural Science Foundation of China (grants 81771092 and 81970910), Jiangsu Province Capability Improvement Project through Science, Technology and Education‐Jiangsu Provincial Research Hospital Cultivation Unit (YJXYYJSDW4) and Jiangsu Provincial Medical Innovation Center (CXZX202227).

## CONFLICT OF INTEREST STATEMENT

The authors declare no conflicts of interest.

## Supporting information


**Data S1.** Supporting information.

## Data Availability

The datasets used and analyzed during the current study are available within the manuscript and its additional files.
