# Supplementary material for: Impaired autophagy‐mediated macrophage polarization contributes to age‐related hyposalivation
Source: Cell Prolif. 2024 Jul 14;57(12):e13714. doi: 10.1111/cpr.13714 (PMC11628751; doi:10.1111/cpr.13714)
Supplement: Supplementary file 1 — Data S1. Supporting information. [file CPR-57-e13714-s001.docx]

**
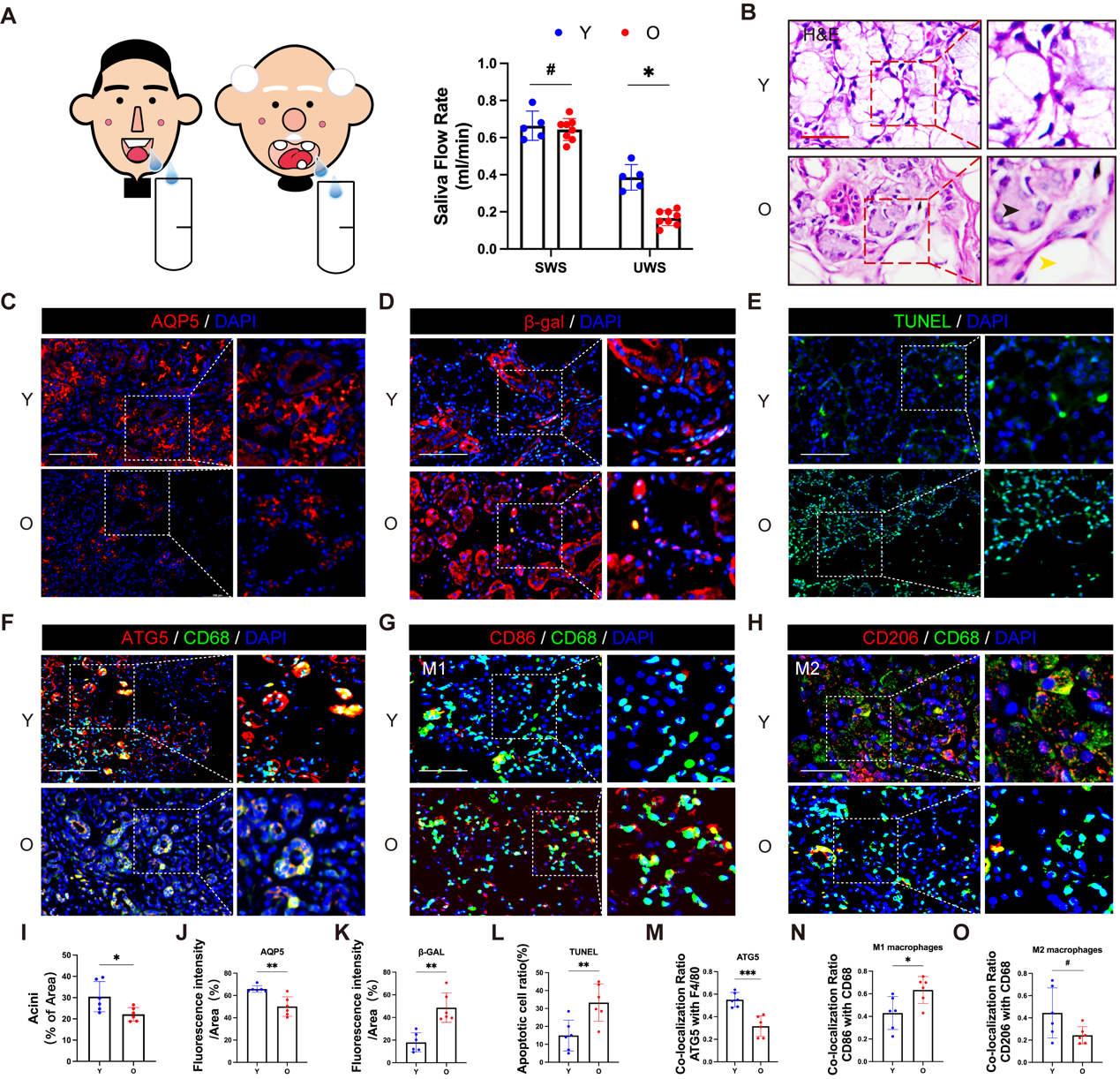
Figure S1. Hyposalivation and age-related histological characterization of SGs in the elderly. (A)** Comparison of salivary flow rate (SFR; ml/min) in unstimulated whole saliva (UWS) and stimulated whole saliva (SWS) between the young (Y; n=5) and the aged (O; n=8) human group. **(B)** Representative images of H&E staining exhibited aging changes in SG structure in humans. Black arrowhead indicated atrophic acini. Yellow arrowhead indicated adipose tissue. n=6. Bar: 400 μm. **(C)** Immunofluorescence staining showed the expression of AQP5 in salivary gland (red: Aquaporins 5 [AQP5], blue: 4’6-diamidino-2-phenylindole [DAPI]). n=6. Bar: 100 μm. **(D)** IHF is performed on SG tissue where Anti-β-galactosidase (β-gal) antibodies that represent senescent cells are used. n=6. Bar: 100 μm. **(E)** The apoptosis cells were significantly increased in elderly SG tissue, as shown by the TUNEL assay. n=6. Bar: 100 μm. **(F)** Expression of autophagy in macrophage was confirmed by CD68 (green), ATG5 (red), and DAPI (blue) immunostaining. n=6. Bar: 200 μm. **(G-H)** Immunofluorescence staining of SG tissues, in which CD68 positive represents macrophages, CD86 positive represents M1 macrophages, and CD206 positive represents M2 macrophages. n=6. Bar: 200 μm. **(I-O)** Quantification of Acini area, Mean intensity of AQP5, Percent of TUNEL positive apoptotic cells, Mean intensity of β-gal, Pearson's coefficient of ATG, CD86, CD206 and CD68. The results were presented as means ± S.D by unpaired 2-tailed Student’s t-test. **p* < 0.05; ***p* < 0.01, ****p* < 0.001; #*p* > 0.05.


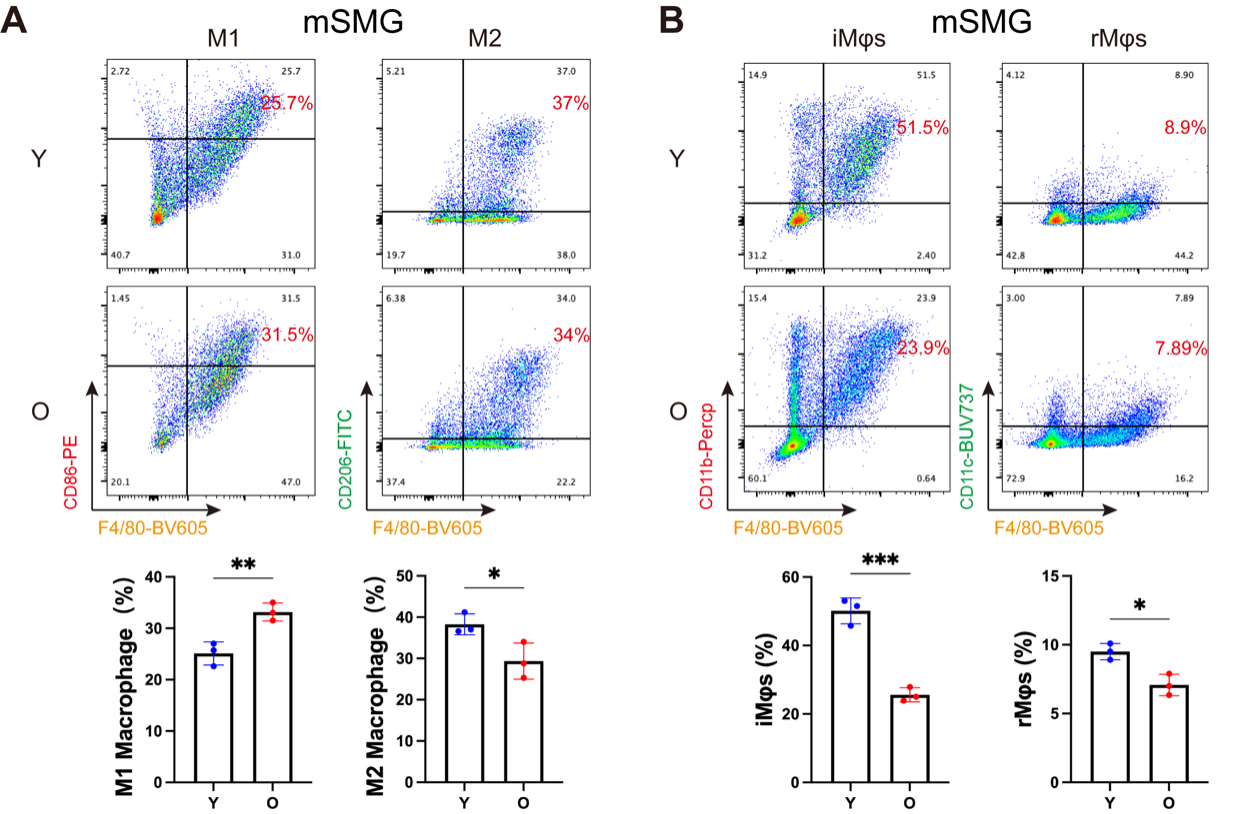


**Figure S2. Polarization characteristics and types of macrophages in submandibular gland. (A)** The proportion of M1 macrophages and M2 macrophages in salivary gland tissue of mice was observed by flow cytometry. n=3. **(B)** Characterization of the tissue resident macrophages (rMφs) and and the infiltrating macrophages (iMφs) in salivary gland using flow cytometry. n=3. The results were presented as means ± S.D by unpaired 2-tailed Student’s t test. **p* < 0.05; ***p* < 0.01, ****p* < 0.001.


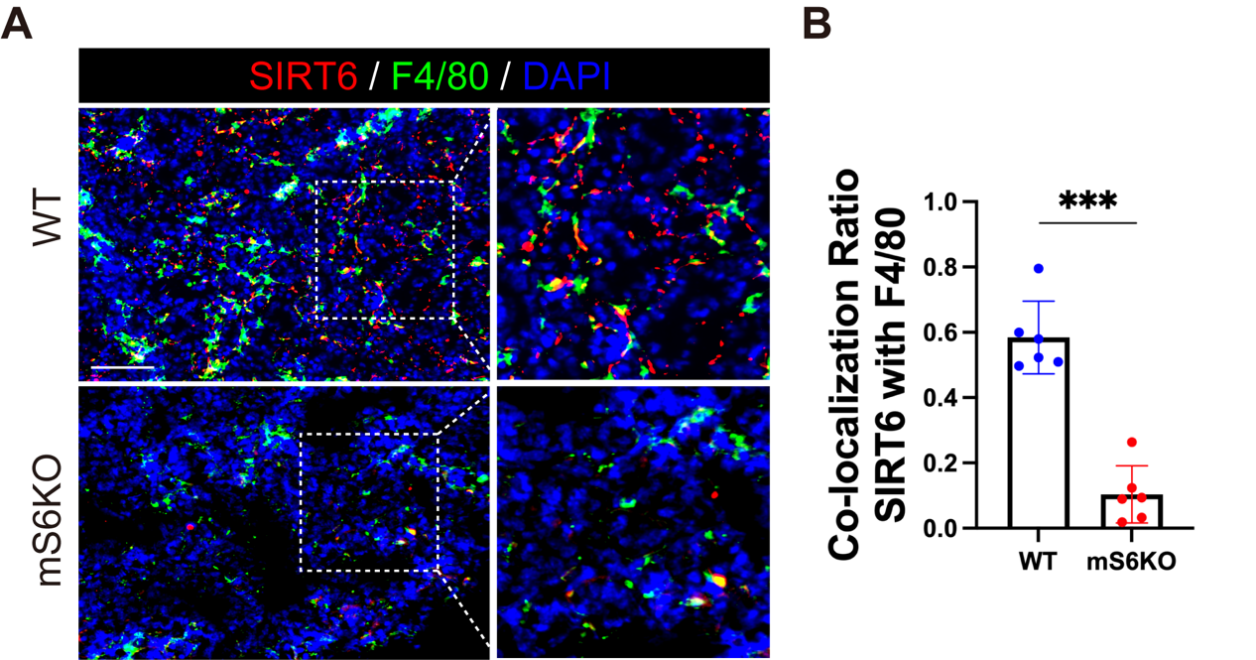


**Figure S3. Deficiency of SIRT6 expression in macrophages of mS6KO mice.** **(A)** Expression of SIRT6 (red) in F4/80 (green) positive cells in the SG tissue of WT and mS6KO mice. n=6. Bar: 100 μm. **(B)** Quantification of Pearson's coefficient of SIRT6 and F4/80. The results were presented as means ± S.D by unpaired 2-tailed Student’s t-test. ****p* < 0.001.


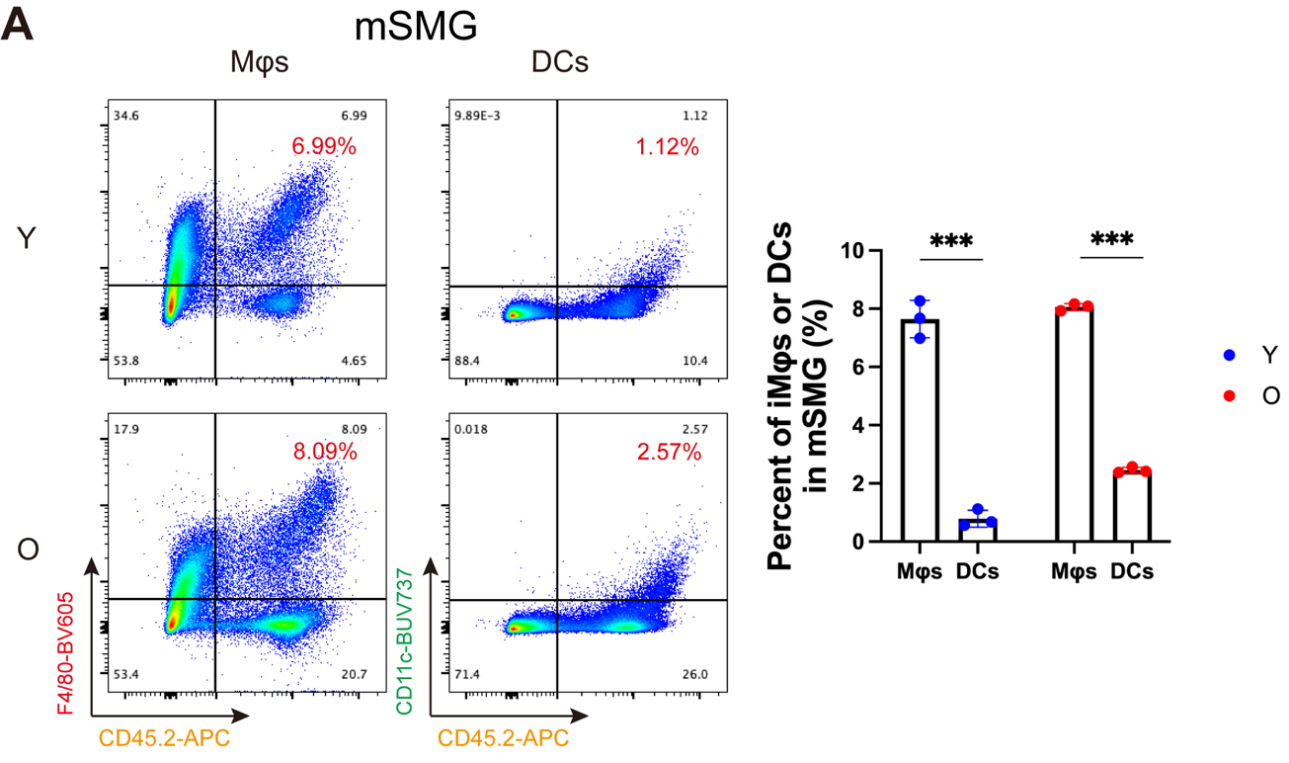


**Figure S4. Percent of macrophages and dendritic cells in mSMG of mice. (A)** The proportion of macrophages and Dendritic cells (DCs) in salivary gland tissue of mice was observed by flow cytometry. n=3. The results were presented as means ± S.D by unpaired 2-tailed Student’s t-test. ****p* < 0.001.

**
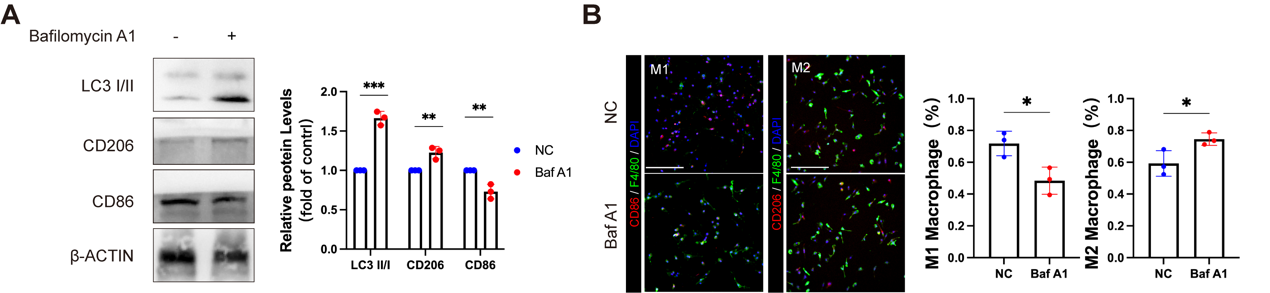
 Figure S5. Baf A1 upregulates autophagy and M2 macrophages polarization.** (A) Immunocyte fluorescence for M1 (CD86) and M2 (CD206) macrophage markers with Baf A1 treatment. n=3. Bar: 200 μm. **(B)** Western blot analysis showing LC3 II/I, ATG5, CD86, CD206, and β-ACTIN in the BMDMs after Baf A1 treatment. n=3. The results were presented as means ± S.D by unpaired 2-tailed Student’s t-test. **p* < 0.05; ***p* < 0.01, ****p* < 0.001.


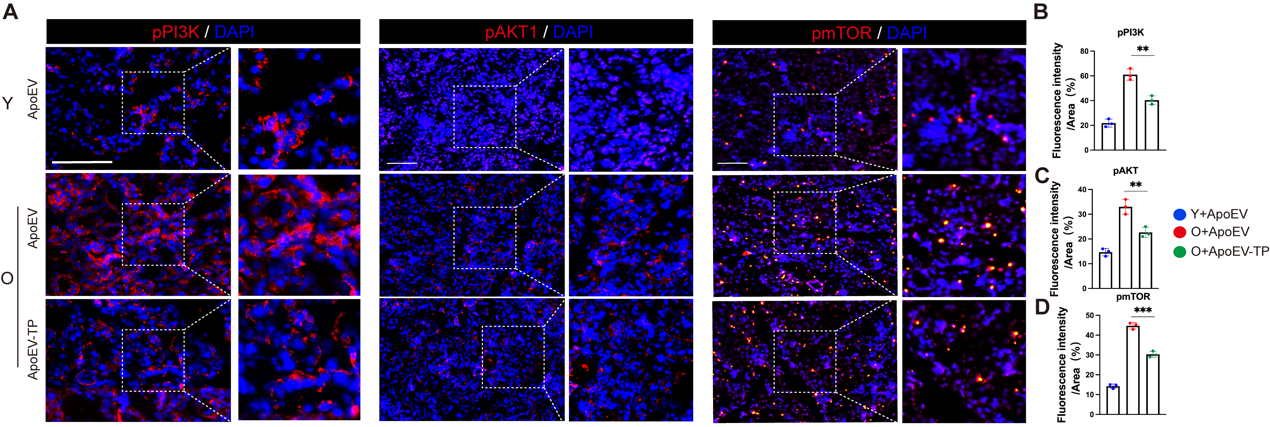


**Figure S6. The phosphorylation levels of PI3K, AKT1, and mTOR in aged SGs declined after the TP-loaded ApoEV treatment. (A)** Immunofluorescent microscopy of SG sections for pPI3K, pAKT1, pmTOR in each group (young mice with ApoEVs treatment [Y+ApoEV]; old mice with ApoEVs treatment [O+ApoEV]; old mice with TP-loaded ApoEVs treatment [O+ApoEV-TP]). n=3. Bar: 100 μm. **(B-D)** Quantification of mean intensity of pPI3K, pAKT1, pmTOR. The results were presented as means ± S.D by unpaired 2-tailed Student’s t-test. ***p* < 0.01, ****p* < 0.001.


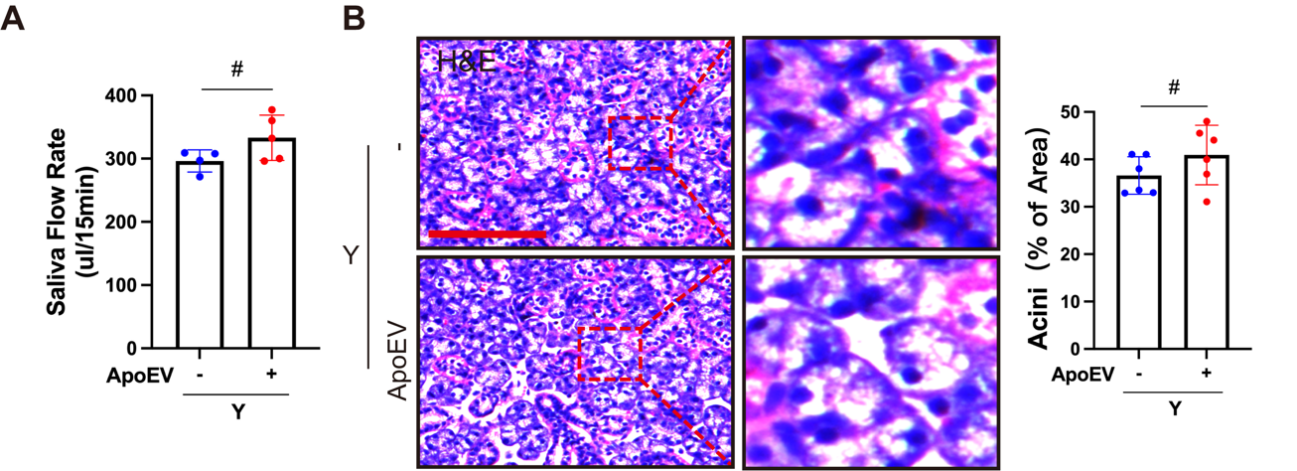


**Figure S7. ApoEV treatment did not enhance salivary gland secretion function in young mice. (A)** Changes of SFR (μl/min) in the young (Y; n=4) and the young mice with ApoEV treatment group (Y+ApoEV; n=5). **(B)** The histological section stained with H&E showed the no significant difference changes in the SG structure of mice. n=6. Bar: 100 μm. Quantification of Acini area. The results were presented as means ± S.D by unpaired 2-tailed Student’s t-test. #*p* > 0.05.

| **Supplementary Table S1:** Inclusion and exclusion criteria of human subject | | | | | | | | | |
| --- | --- | --- | --- | --- | --- | --- | --- | --- | --- |
| **Category** | **Exclusion Criteria** | | | | | | | |  |
| **Oral diseases** | Infections/ulcers/herpes/spots/allergic diseases in oral mucosal | | | | | | | | … |
|  | Inflammatory diseases of the salivary glands | | | | | | | |  |
| **Endocrine diseases** | Adrenocortical diseases | | | | Hypertension | | | | … |
|  | Diabetes mellitus | | | | Thyroid diseases | | | |  |
| **Autoimmune**  **diseases** | Sjögren syndrome (SS) | | | | Rheumatoid arthritis (RA) | | | | … |
|  | Autoimmune thyroid diseases | | | | Scleroderma | | | |  |
|  | Primary biliary cirrhosis | | | | Systemic lupus | | | |  |
|  | Erythematosus | | | | | | | |  |
| **Infectious causes** | Actinomycosis | | | | Human immunodeficiency virus | | | | … |
|  | Hepatitis C virus | | | | Epstein-Barr virus | | | |  |
|  | Giant cell disease berry | | | | Cytomegalovirus | | | |  |
|  | Upper Respiratory  Tract Infections (URTI) | | | | Human T-lymphotropic  virus type 1 virus | | | |  |
| **Granulomatous causes** | Tuberculosis | | | | Sarcoidosis | | | | … |
| **Mental illness** | Mental anxiety | | | | Depression | | | | … |
| **Other systemic causes** | End-stage renal disease | | | | Parkinson disease | | | | … |
|  | Hemochromatosis | | | Ectodermal dysplasia | | | | Amyloidosis |  |
|  | Chronic graft-versus-host disease after stem cell transplantation | | | | | | | |  |
| **Medications** | Anticlonus drugs | Antidepressants | | | | Anticholinergic agents | | | … |
|  | Antihistamines | Antihypertensives | | | | Sedative agents | | |  |
|  | Anti-HIV drugs | Cytotoxic drugs | | | | Antineoplastic drugs | | |  |
|  | Opioids | Diuretics | | | | Antipsychotics | | |  |
|  | Antiparkinsonian medications | | | | | | | |  |
| **Lifestyle factors** | Tobacco use | Alcohol use | | | | Dehydration | | | … |
|  | Heavy snoring | Mouth breathing | | | | Sleep apnea syndrome | | |  |
|  | Caffeinated beverage consumption | | | | | Insomnia | | |  |
| **Hematologic tumor** | Leukemia | | lymphoma | | | | multiple myeloma | | … |
| **Salivary gland tumor** | Pleomorphic adenoma | | mucoepidermoid carcinoma | | | | adenoid cystic carcinoma | | … |
| **Head and neck radiation** |  | | | | | | | | … |
| **Salivary glands were removed after trauma or hand wood** |  | | | | | | | | … |
| **Category** | Inclusion **Criteria** | | | | | | | |  |
| **Gender** | male | | | | | | | |  |
| **Age** | 25-35y（Young group）  65-85y（Old group） | | | | | | | |  |

| **Supplementary Table S2:** **Data of antibodies used in our research** | | | | | |
| --- | --- | --- | --- | --- | --- |
| **Antibody** | **WB** | **IHF** | **ICC** | **Specificity** | **Company** |
| CD68  (Ab955) | - | 1:50 | - | Mouse | Abcam |
| F4/80  (Ab6640) | - | 1:100 | 1:100 | Rat | Abcam |
| CD86  (DF6332) | 1:1000 | 1:100 | 1:100 | Rabbit | Affnity |
| CD206  (187041-AP) | 1:100 | 1:1000 | 1:100 | Rabbit | Proteintech |
| ATG5  (10181-2-AP) | 1:1000 | 1:100 | - | Rabbit | Proteintech |
| LC3 A  (#4599) | 1:1000 | - |  | Rabbit | Cell Signaling Technology |
| β-ACTIN  (81115-1-RR) | 1:10000 | - | - | Rabbit | Proteintech |
| SIRT6  (Ab119007) | 1:1000 | - | - | Mouse | Abcam |
| β-galactosidase  (Ab203749) | - | 1:100 | - | Rabbit | Abcam |
| CD9  (60232-1-Ig) | 1:5000 | - | - | Mouse | Proteintech |
| CD81  (27855-1-AP) | 1:1000 | - | - | Rabbit | Proteintech |
| TSG101  (28283-1-AP) | 1:5000 | - | - | Rabbit | Proteintech |
| Calnexin  (10427-2-AP) | 1:5000 | - | - | Rabbit | Proteintech |
| Cleaved Caspase-3  (#9661) | 1:1000 | - | - | Rabbit | Cell Signaling Technology |
| AKT  (#2920) | 1:2000 | - | - | Mouse | Cell Signaling Technology |
| p-AKT  (MB10435) | 1:2000 | - | - | Rabbit | Bioworld |
| PI3K  (AF6241) | 1:1000 | - | - | Rabbit | Affnity |
| p-PI3K  (ABP50495) | 1:800 | - | - | Rabbit | Abbkine |
| mTOR  (#2983S) | 1:1000 | - | - | Rabbit | Cell Signaling Technology |
| pmTOR  (#5536S) | 1:1000 | - | - | Rabbit | Cell Signaling Technology |
| AQP5  (20334-1-AP) | - | 1:3000 | - | Rabbit | Proteintech |
| APC anti-mouse CD45.2 Antibody  (109813) |  |  |  | Mouse | Biolegend |
| PerCP-Cy^TM^5.5 Rat Anti-CD11b Antibody  (561114) |  |  |  | Rat | BD Pharmingen |
| BUV737 Hamster Anti-mouse CD11c Antibody  (612797) |  |  |  | Rat | BD Pharmingen |
| Brilliant Violet 605™ anti-mouse F4/80 Antibody  (123133) |  |  |  | Rat | Biolegend |
| PE Rat Anti-mouse CD86 Antibody  (561963) |  |  |  | Rat | BD Pharmingen |
| FITC Anti-mouse CD206 (MMR) Antibody  (141703) |  |  |  | Rat | Biolegend |
| HRP-conjugated Affinipure Goat Anti-Rabbit IgG(H+L)  (SA00001-2) | 1:8000 | - | - | Goat | Proteintech |
| HRP-conjugated Affinipure Goat Anti-Mouse IgG(H+L)  ( SA00001-1) | 1:8000 | - | - | Goat | Proteintech |
| Fluorescein (FITC)–conjugated Affinipure Goat Anti-Mouse IgG(H+L) | - | 1:50 | 1:50 | Goat | Proteintech |
| Fluorescein (FITC)–conjugated Affinipure Goat Anti-Rat IgG(H+L)  (SA00003-11) | - | 1:50 | 1:50 | Goat | Proteintech |
| Cy3–conjugated Affinipure Goat Anti-Rabbit IgG(H+L)  (SA00009-2) | - | 1:100 | 1:100 | Goat | Proteintech |

| **Supplementary Table S3:** **Data of reagents used in cell experiments** | |
| --- | --- |
| **Reagents** | **Company** |
| 1. Methyladenine   (HY-19312) | MCE |
| Rapamycin  (HY-10219) | MCE |
| OSS_128167  (S8627) | Selleck |
| 1. CSF   (416-ML-050/CF) | R&D Systems |
| Celltracker CM-DiI  (40718ES50) | Yeasen |
| Bafilomycin A1 (BafA1) | ThermoFisher |

**Supplementary materials and methods**

**Measurement of salivary flow rate in human**

Due to the rhythm of saliva secretion, saliva samples from people were collected between 9:00 am and 11:00 am every day. Explain the saliva collection process to the human subject in advance to ensure good compliance. Before the test, all subjects did not smoke, eat or drink any liquids other than water, and did not perform any special oral hygiene except gargling with water to avoid affecting the test results. Before collecting saliva, the subjects were asked to gargle with water and rest quietly for 5min before collecting. To collect saliva, the subject should be in a quiet, comfortable, well-lit environment, sitting position, eyes open, head slightly forward; Ask subjects to turn their heads and place a 50mL centrifuge tube in the corner of their mouth to allow the saliva to flow naturally without coughing up mucus. During saliva collection, the subject is asked not to imagine irritating food, and not to suck or swallow saliva until the end. The whole period lasted for 5min, and the whole process of saliva collection was supervised. At the end of the period, the subjects spit all the remaining saliva in their mouths into the centrifuge tube. To collect stimulated saliva, 2-3 drops of citric acid were applied to the dorsum of the tongue with a dropper, and samples were spat in graded disposable plastic cups for at least 5 min. The unstimulated and stimulated saliva volumes were recorded and the salivary flow rate was expressed in mL/min.

**Flow cytometry**

The mouse submandibular gland tissue was chopped with a blade and digested with IV type collagenase (1mg/ml) and 5mM CaCl_2_ solution in 37°C for 1 hour, and then filtered through a 70mm filter to obtain a single-cell suspension. Then the single-cell suspension stained with the following antibodies: APC anti-CD45.2, PerCP-Cy^TM^5.5 anti-CD11B, BUV737 anti-CD11C, BV605 anti-F4/80, PE anti-CD86, FITC anti-CD206. FlowJo v.10 software was used for the data analysis.
